# Lower Limb Progressive Resistance Training Improves Leg Strength but Not Gait Speed or Balance in Parkinson’s Disease: A Systematic Review and Meta-Analysis

**DOI:** 10.3389/fnagi.2015.00040

**Published:** 2015-03-24

**Authors:** Alex Tillman, Makii Muthalib, Ashlee M. Hendy, Liam G. Johnson, Timo Rantalainen, Dawson J. Kidgell, Peter G. Enticott, Wei-Peng Teo

**Affiliations:** ^1^Centre for Physical Activity and Nutrition Research, School of Exercise and Nutrition Sciences, Deakin University, Burwood, VIC, Australia; ^2^Movement to Health (M2H) Laboratory, Euromov, University of Montpellier, Montpellier, France; ^3^The Florey Institute of Neuroscience and Mental Health, University of Melbourne, Heidelberg, VIC, Australia; ^4^Institute of Sport, Exercise, and Active Living (ISEAL), Victoria University, Melbourne, VIC, Australia; ^5^Department of Rehabilitation, Nutrition and Sport, School of Allied Health, La Trobe University, Melbourne, VIC, Australia; ^6^Cognitive Neuroscience Unit, School of Psychology, Deakin University, Burwood, VIC, Australia

**Keywords:** Parkinson’s disease, progressive resistance training, leg strength, gait speed, balance

## Abstract

The use of progressive resistance training (PRT) to improve gait and balance in people with Parkinson’s disease (PD) is an emerging area of interest. However, the main effects of PRT on lower limb functions such as gait, balance, and leg strength in people with PD remain unclear. Therefore, the aim of the meta-analysis is to evaluate the evidence surrounding the use of PRT to improve gait and balance in people with PD. Five electronic databases, from inception to December 2014, were searched to identify the relevant studies. Data extraction was performed by two independent reviewers and methodological quality was assessed using the PEDro scale. Standardized mean differences (SMD) and 95% confidence intervals (CIs) of fixed and random effects models were used to calculate the effect sizes between experimental and control groups and *I*^2^ statistics were used to determine levels of heterogeneity. In total, seven studies were identified consisting of 172 participants (experimental *n* = 84; control *n* = 88). The pooled results showed a moderate but significant effect of PRT on leg strength (SMD 1.42, 95% CI 0.464–2.376); however, no significant effects were observed for gait speed (SMD 0.418, 95% CI −0.219 to 1.055). No significant effects were observed for balance measures included in this review. In conclusion, our results showed no discernable effect of PRT on gait and balance measures, although this is likely due to the lack of studies available. It may be suggested that PRT be performed in conjunction with balance or task-specific functional training to elicit greater lower limb functional benefits in people with PD.

## Introduction

Parkinson’s disease (PD) is a progressive neurodegenerative movement disorder, which stems primarily from the death of dopaminergic (DA) neurons in the basal ganglia circuitry, and is characterized by motor abnormalities such as resting tremors, bradykinesia, rigidity, and postural instability (Hornykiewicz and Kish, 1987; Yoritaka et al., 2013). In addition to motor deficits, people with PD often experience non-motor impairments such as cognitive, neuropsychiatric, sleep, autonomic and sensory disturbances that results in a reduced quality-of-life (Chaudhuri et al., 2006; Park and Stacy, 2009). It has been estimated that approximately 7 million people suffer from PD worldwide and these figures are expected to increase as the average age of the population increases (Kasten et al., 2007).

Due to the neurological impairments associated with PD, coupled with age-related musculoskeletal declines, people diagnosed with PD are at greatest risk of falls with several studies reporting 38–87% of all people with PD experiencing severe falls at least once in their lifetime (Ashburn et al., 2001; Balash et al., 2005). A meta-analysis of several prospective studies further showed that the rate of recurrent falling over a 3-month period was 57% among people with PD who had reported previous falls (Pickering et al., 2007). Given the severity and chronic nature of PD, DA medications are often initiated during the early stages of the disease and continue to be prescribed throughout their lives (Stowe et al., 2008; Grosset et al., 2010). However, it is estimated that the annual medical cost associated with PD is between $10,043 and $12,491 (Noyes et al., 2006; Boland and Stacy, 2012) and the long-term use of DA medication is not without side-effects, which may include addiction, behavioral disturbances (Merims and Giladi, 2008), and levodopa-induced dyskinesia (Bezard et al., 2001). More importantly, studies have shown that the underlying mechanisms of postural instability and gait difficulties in PD are dopamine-resistant (Bloem et al., 1996; Bohnen and Cham, 2006), which may explain the greater fall rate in people with PD (Johnson et al., 2013).

In recent years, the use of exercise to improve motor symptoms in PD has received great interest. Several meta-analyses investigating the effects of aerobic exercise interventions such as treadmill walking reported significant improvements to gait, balance, and cardiovascular fitness in people with moderate-to-severe PD (Goodwin et al., 2008; Shu et al., 2014). In particular, several studies have also reported beneficial effects on motor function, muscle strength, and endurance following progressive resistance training (PRT) (Brienesse and Emerson, 2013; Lima et al., 2013). Recently, Corcos et al. (2013) conducted a large randomized controlled trial investigating the effects of a 24-month whole-body PRT intervention in 38 PD participants and showed a clinically significant improvement in off-medication unified Parkinson’s disease rating scale (UPDRS) scores. Although it is unclear as to what mechanisms underpin the improvements in motor symptoms following PRT, several studies suggest that PRT may help to improve muscle strength and mass (Hirsch et al., 2003; Dibble et al., 2006, 2009), and normalize neuroplasticity that may otherwise be impaired in people with PD (Teo et al., 2014).

Despite the evidence supporting the use of PRT to improve clinical measures of motor function, little is known about the effects of PRT on gait and balance measures in people with PD. Several meta-analyses conducted on the effects of PRT on gait and balance measures in healthy aging population showed inconsistent findings due to the wide disparity in the type of measures used (Latham et al., 2004; Orr et al., 2008; Steib et al., 2010). However, preliminary studies in people with mild-to-moderate PD found that high-intensity eccentric lower limb resistance training on a cycle ergometer resulted in increased muscle size in the quadriceps muscle, which correlated to improvements in lower limb muscle force and measures of mobility (Dibble et al., 2006, 2009). However, it is unclear if PRT alone is able to elicit improvements to balance and gait in people with PD. To the best of our knowledge, only one other meta-analysis conducted by Lima et al. (2013) indicated that PRT is beneficial for improving leg strength and physical function in people with PD that is not limited to just gait and balance. Therefore, this meta-analysis aims to evaluate the current literature for evidence to support the functional benefits of PRT on gait and balance in people with PD and to identify critical gaps in the literature that needs to be addressed in future research.

## Methods

### Search strategy

**Figure 1:**
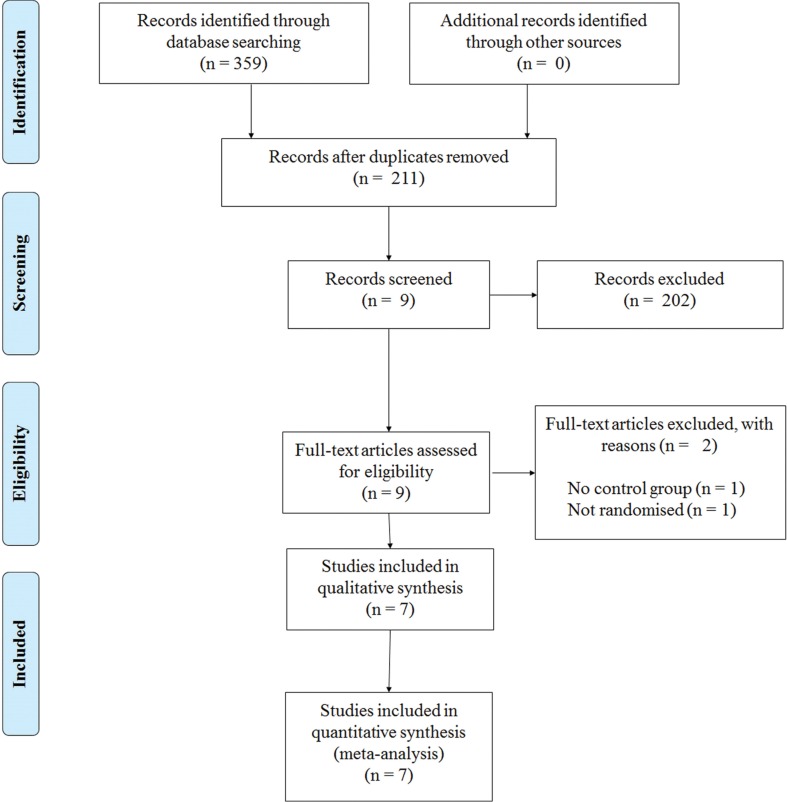
**PRISMA flow chart for the selection of studies included in this meta-analysis**.

This review has been informed by the PRISMA statement (Moher et al., 2009). The following electronic databases were searched from their inception to December 2014: PubMed, MEDLINE, PsycINFO, Embase, and Scopus. The following keywords were used in combinations: Parkinson, Parkinson’s disease, Parkinsonism, resistance training, strength training, exercise, gait, balance, and physical therapy. Figure 1 shows a flow diagram of the processing of search results from initial searches to the final included studies.

### Selection criteria

Studies were included if (1) the aim of the study was to examine the effects of PRT on gait and balance in PD; (2) the target population was aged between 20 and 85 years with a confirmed diagnosis of PD; (3) the main intervention was PRT and the exercises specifically targeted the lower limb; (4) the effects of the resistance exercise intervention were compared to other training intervention, including other forms of exercise or physical activity; (4) the outcomes included either balance, gait, or leg strength. A study was excluded if (1) the effects of other forms of exercise not involving PRT were evaluated (such as behavioral interventions, music therapy, cueing strategies, or whole body vibration); (2) does not have a control group; (3) it was not randomized.

Data was extracted by two independent reviewers (Alex Tillman and Wei-Peng Teo) and is summarized in Table 1. To determine the eligibility of each study, the title and abstract was screened independently, and if the title and abstract did not meet the inclusion criteria, it was excluded. Only full-text articles that were obtainable were used and if the article did not report the relevant information, the corresponding authors were contacted. Further eligibility screening was then independently conducted for these articles by two reviewers (Alex Tillman and Ashlee M. Hendy), by using a standardized form containing the details of the inclusion criteria. For crossover studies, only the results from the first phase of the study were used to prevent any biased implications when interpreting the results. Any discrepancies were resolved by consensus with reference to the original article.

**Table 1:** **Characteristics of studies of PRT in people with PD**.

Study	Sample size, mean age (years)	Hoehn and Yahr stage	Mean duration of PD (years)	Intervention duration (weeks)	Main outcome assessments	Intervention group	Control group
Shulman et al. (2013), US	*n* = 67; 65.8 (SD 10.7)	1–3	6.2 (SD 3.8)	16	• Gait speed	Lower limb resistance training (2 sets × 10 repetitions)	Treadmill training
					• Cardiovascular fitness	• Leg presses	
					• muscle strength	• Leg extensions	
					• UPDRS	• Leg curls	
Hass et al. (2012), US	*n* = 18; 65.5 (SD 2.1)	1–3	8.7 (SD 3.3)	10	• COP displacement	Lower limb resistance training (2 sets × 10–12 repetitions)	No intervention
					• Gait initiation	• Seated leg presses	
					• Stride length and velocity	• Seated knee extension	
						• Seated knee flexion	
						• Abdominal curls	
						• Back extension	
						• Seated calf raises	
						• Multi-directional seat ankle movements with theraband	
Allen et al. (2010), AU	*n* = 48; 67 (SD 1.4)	NR	8 (SD 1.4)	24	• UPDRS	PRT (40–60 min)	No intervention
					• PDQ-39	• High stepping on the spot	
					• Strength	• Standing with a decreased base	
					• Balance	• Graded reaching in standing	
					• Freezing	• Stepping in different directions	
					• Postural sway	• Walking	
						• Sit-to-stand	
						• Heel raises	
						• Lateral step-up	
						• Forward step-up	
						• Half-squats sliding down a wall	
						Balance training (10 s × 15 reps each leg)	
						• Standing on one leg	
Schilling et al. (2010), US	*n* = 18; 59.1 (SD 3.0)	1–2.5	NR	8	• Strength	Lower limb resistance training (3 sets × 5–8 repetitions)	Standard exercise management of PD.
					• Gait function	• Leg press	
					• ABC	• Leg curl	
						• Calf press	
Dibble et al. (2009), US	*n* = 19; 65.6 (SD 1.9)	2.5	6.3 (SD 0.2)	12	• Muscle force production	Lower limb and upper limb resistance training (45–60 min, 3 days/week)	Standard exercise management of PD.
					• Quality of life	• Stretching	
					• UPDRS	• Walking (treadmill)	
					• Gait speed	• Riding (cycle ergometer)	
					• PDQ-39	• Machine and free weights (upper extremities)	
						Muscle force (3 sets × 50–75% of perceived maximal effort)	
						• 60° seated fixed knee flexion	
Dibble et al. (2006), US	*n* = 19; 65.6 (SD 1.9)	2.5	6.3 (SD 0.2)	12	• Muscle endurance	Lower limb resistance training (45–60 min 3 days/week)	Standard exercise management of PD.
					• Flexibility	• Light calisthenics and stretching	
					• Balance	• Walking (treadmill)	
					• Muscle production	• Riding (cycle ergometer)	
					• UPDRS	• Lifting weights (machine and free weights)	
Hirsch et al. (2003), US	*n* = 15; 73.2 (SD 3.4)	1–2	6.9 (SD 1.9)	10	• Balance	Lower limb resistance training (60% 4-RM, 1 set × 12 repetitions)	No intervention
					• Muscle strength	• Moving legs simultaneously at 6–9 s pre repetition	
						Lower limb muscle strength (standardized weight-and-pulley system, 4-RM, 1 set × 4 repetitions)	
						• Knee extension	
						• Knee flexion	
						• Ankle plantarflexion	
						Balance training (computerized dynamic posturograph)	
						• Sensory orientation test	

*NR, not reported; PRT, progressive resistance training; TBE, traditional balance training; COMBI, combination of both; UPDRS, unified Parkinson’s disease rating scale; GI, gait initiation; COP, center-of-pressure; PD, Parkinson’s disease; ABC, activities-specific balance confidence; PDQ-39, Parkinson’s disease questionnaire 39*.

### Quality assessment of studies

For each article, one reviewer (Alex Tillman) extracted all the data and a second reviewer (Wei-Peng Teo) performed a secondary check on the extracted data set. Any discrepancies found were resolved by discussion and care was taken to identify duplicate reports of any articles found. Only published articles that include articles *in press* were included in the meta-analysis. The methodological quality of each study was assessed independently by two reviewers (Dawson J. Kidgell and Timo Rantalainen) separate from the data extraction phase using the physiotherapy evidence database (PEDro) scale (ranging from 0–10 points). The PEDro scale is an assessment tool for evaluating methodological quality of randomized control trials conducted in the field of physiotherapy and has been previously shown to have fair-to-good reliability (Maher et al., 2003; Macedo et al., 2010). A cut-off point of 6 on the PEDro scale was used to indicate high-quality studies (Macedo et al., 2010). Any disagreements in scores were resolved by discussion between reviewers, with the judgment of the primary author (Wei-Peng Teo) being sought if necessary. All scores assigned to each study were agreed upon by consensus and are presented in Table 2.

**Table 2:** **PEDro scale of quality for eligible randomized controlled trials**.

Study	Random allocation	Concealed allocation	Similar at baseline	Subjects blinded	Therapists blinded	Assessors blinded	<15% Dropouts	Intention-to-treat analysis	Between-group comparisons	Point measures and variability data	Total
Shulman et al. (2013), US	1	0	1	0	0	0	1	0	1	1	5
Hass et al. (2012), US	1	0	1	0	0	0	1	0	1	1	5
Allen et al. (2010), AU	1	1	1	0	0	1	1	1	1	1	8
Schilling et al. (2010), US	1	0	1	0	0	0	1	0	1	1	5
Dibble et al. (2009), US	0	0	1	0	0	0	1	0	1	1	4
Dibble et al. (2006), US	0	0	1	0	0	0	1	0	1	1	4
Hirsch et al. (2003), US	1	0	1	0	0	0	1	0	1	1	5

*Each criterion was scored as either 1 or 0 according to whether the criteria were met or not respectively; PEDro, physiotherapy evidence database*.

### Selection of outcome measures

The outcome measures for balance, gait speed, and leg strength used in the meta-analysis were selected based on their ability to provide direct quantitative measures or confidence scores. Clinical outcome measures such as the 6min and 10 m walking tests were used to provide a measure of gait speed. Additionally, studies using biomechanical analysis such as the Walkway and 3-dimensional motion capture system that provided a quantitative measure of gait speed were also included. Balance is a subjective measure and can be quantified in several ways. Studies that include direct posturographic measures using forceplates. Swaymeters attached to the participant’s waist or sensory orientation tests were included into our meta-analysis; however, studies that provided confident scores of balance during activity-specific tasks were also included. Lastly, outcome measures that provided a direct measure of leg strength in Newtons or kilograms such as 1-repetition maximum leg presses, force dynamometers or strain gages attached to the lower limb were included into the meta-analysis.

### Data synthesis and analysis

Random effects meta-analyses were conducted with MedCalc Statistical Software v14.12.0 (MedCalc Software bvba, Ostend, Belgium; http://www.medcalc.org; 2014). Hedges’ *g* was used to measure the effect sizes for all meta-analyses and presented as standardized mean difference (SMD) and 95% confidence intervals (CI). Heterogeneity across studies was tested based on *I*^2^ statistics. Studies with *I*^2^ <40% was considered to have low heterogeneity, *I*^2^ = 40–75% was considered moderate heterogeneity, and *I*^2^ >75% was considered to have high heterogeneity. Fisher’s method of combining *p*-values was applied to test for overall pooled effects for each outcome measure.

## Results

### Study selection

Our initial search yielded 359 references. Following screening of the title and abstract and removal of duplicates, nine studies were further screened. After assessments against our inclusion criteria, two studies were removed and seven studies were kept for further analyses (Figure 1).

### Participant characteristics

In total, 172 people with PD were assessed across seven studies. The mean age for all participants was 66 ± 3.5 years, while the mean duration of PD was 7.1 ± 1.8 years. All seven studies recruited participants with mild-to-moderate motor disabilities as identified by the Hoehn & Yahr scale of 1–3.

### Methodological quality

Table 2 highlights the scores for each criterion using the PEDro scale. It was determined that the average score for all seven trials was five (lowest four, highest eight). Across the seven studies used, it was found that all participants and therapists administering the program were not blinded to the treatment of participants. Only one study concealed the allocation of all participants, used blinded assessors, and employed intention-to-treat analysis (Allen et al., 2010).

### Gait speed

**Figure 2:**
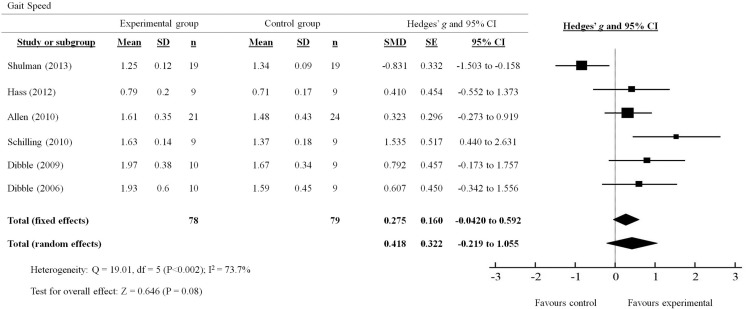
**Forest plot showing the effects of progressive resistance training on gait speed in people with PD**.

**Figure 3:**
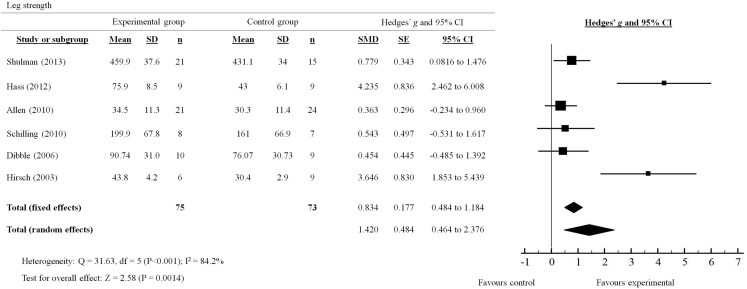
**Forest plot showing the effects of progressive resistance training on leg strength in people with PD**.

A total of six out of seven studies reviewed presented quantitative measures of gait speed (Figure 2). The effects of PRT on gait speed were examined by pooling post-intervention data from the six studies (experimental *n* = 78; control *n* = 79; Figure 3). Overall, the pooled results suggest improvement in gait speed following PRT was not statistically significant (total random effects = SMD 0.418, 95% CI −0.219 to 1.055). Only Schilling et al. (2010) demonstrated a favorable effect for the use of PRT to improve gait speed (SMD 1.535, 95% CI 0.440–2.631), while Shulman et al. (2013) showed a favorable effect for the control group instead (SMD −0.831, 95% CI −1.503 to −0.158).

### Balance

Out of the seven studies reviewed, only three studies provided either direct or indirect measures of the effects of PRT on balance in people with PD (Table 3). Overall, the MD across the three studies suggest a non-statistically significant improvement in balance measures following PRT. Although both studies by Allen et al. (2010) and Schilling et al. (2010) did demonstrate a trending improvement following PRT, no significant effects were observed between experimental and control groups in either studies. Only Hirsch et al. (2003) showed favorable support for the use of PRT to improve balance, measured by the EquiTest Balance System, over 10 weeks of training (MD 13, 95% CI, 8–18).

**Table 3:** **Effects of PRT on balance measures**.

Trial	Intervention duration (weeks)	Balance measure	Mean difference (MD) between experimental vs control groups	
			MD	95% *CI*
Allen et al. (2010)	24	Static postural sway (standing)	6.8	−36.1 to 49.7
Schilling et al. (2010)	8	Activity-based balance confidence scale	6.7	−8.5 to 21.9
Hirsch et al. (2003)	10	EquiTest balance score	13	8–18

### Leg strength

A total of six out of seven studies reviewed presented quantitative measures of leg strength (Figure 3). The effects of PRT on leg strength were examined by pooling post-intervention data from the six trials (experimental *n* = 75; control *n* = 73). Overall, the results significantly favored (*p* = 0.0014) the use of PRT to improve leg strength in people with PD (total random effects = SMD 1.42, 95% CI 0.464–2.376). In particular, studies by Shulman et al. (2013); Hass et al. (2012) and Hirsch et al. (2003) showed effect sizes that were in favor of the experimental group compared to the control group (Shulman: SMD 0.779, *95*% CI 0.0816–1.476; Hass: SMD 4.235, 95% CI 2.462–6.008; Hirsch: SMD 3.646, 95% CI 1.853–5.439*)*.

## Discussion

The aim of this meta-analysis was to determine if PRT was beneficial to measures of gait, balance, and leg strength in people with PD in comparison to other exercise interventions or no intervention. Our main findings showed non-statistically significant improvements in gait speed and balance measures that are concomitant with a moderate but significant increase in leg strength following an average of 16 weeks of PRT. Based on these findings, we were not able to conclusively determine if PRT was indeed beneficial for improving gait and balance in people with PD.

From our systematic review, we have determined that all studies reported were of sound methodology (average PEDro score = 5) which suggests that our findings were credible. However, only one study (Allen et al., 2010) used intention-to-treat analysis, blinded assessors, and had concealed participant allocation while the rest did not. In addition, both participants and therapists from all seven studies were not blinded to the grouping in which participants were allocated to. This presents as a major limitation in the methodology of the current studies; however, it may also be difficult, particularly in non-pharmacological interventions, to blind both participants and therapists of the grouping in which the participants will receive.

Results from our meta-analysis showed that PRT significantly increased leg strength in people with mild-to-moderate severe PD following PRT lasting from 8 to 24 weeks. The increase in leg strength was expected and is consistent with the results from a recent meta-analysis conducted by Lima et al. (2013). Despite the large variation in training protocols used for PRT between studies (duration: 8–24 weeks; frequency: 2–3sessions/week on non-consecutive days), the intensity for each PRT intervention was approximately 60–80% of one repetition maximum for each exercise (Hirsch et al., 2003; Schilling et al., 2010; Hass et al., 2012) or between 13 and 15 points on the rating-of-perceived exertion scale (Dibble et al., 2006; Allen et al., 2010). Such training intensities are appropriate for strength adaptations even in healthy older adults (>65 years) (Harris et al., 2004; Debeliso et al., 2005) and are likely to support muscle morphology changes. Indeed, Dibble et al. (2006) showed a significant increase in muscle volume in people with mild-to-moderate PD that was correlated with an increase in muscle strength following 12 weeks of high-force eccentric training.

Apart from neuromuscular strength, gait speed and balance are two other important lower limb functions that are often compromised in people with PD. Significant improvements in clinical and functional measures of gait following aerobic exercise, particularly with treadmill walking, were demonstrated in a recent meta-analysis by Shu et al. (2014). It is therefore reasonable to suggest that lower limb PRT may confer similar benefits considering the ability for PRT to improve leg strength. Interestingly, our results did not provide evidence for or against the effects of PRT in improving overall gait speed measured by various walking tests, such as the 6min and 10 m walk tests (Dibble et al., 2006; Shulman et al., 2013), short physical performance battery (Allen et al., 2010), or by 3-dimensional motion analysis (Hass et al., 2012). It is apparent that despite an increase in leg strength following PRT, overall gait speed was not significantly improved. The results from our meta-analysis were similar to those found in healthy aging participants showing little effects of PRT on gait speed and balance (Orr et al., 2008; Steib et al., 2010). Similarly, our findings could not conclusively determine if indeed balance was improved by PRT. Only three studies directly or indirectly measured indices of balance. Allen et al. (2010) directly measured balance using static posturography (sway distance on stable and unstable platforms) and found no appreciable difference between the experimental and control group. Similarly, Schilling et al. (2010) reported no difference in the activity-based balanced scale between experimental and control groups. Only Hirsch et al. (2003) demonstrated a significant effect of PRT on balance using the EquiTest Balance System, which comprises a battery of computerized dynamic posturography measures. The lack of balance measures reported in PRT intervention studies limits our analysis and we suggest a cautious approach is taken when evaluating this data as our findings may not accurately reflect the efficacy of PRT on measures of balance.

In light of our findings, several possible reasons could explain why our meta-analysis did not show any significant improvements in gait speed and balance. The first, and most likely, reason would be that four out of seven studies reviewed used active controls that performed low-intensity balance and treadmill-walking exercises (Hirsch et al., 2003; Dibble et al., 2006, 2009; Shulman et al., 2013). It should be noted that in the four studies that used an active control group, significant improvements between pre and post measures of balance and gait speed were also observed in the PRT intervention groups. Secondly, gait speed is only one of many measures of gait for which PRT may not specifically improve. It may be argued that PRT could have resulted in significant improvements in other gait measures such as gait variability, initiation velocity, stride length, and frequency. Indeed, results from Hass et al. (2012) did show a significant improvement in initial stride velocity following 10 weeks of PRT in mild-to-moderate PD participants. Thirdly, it may also be argued that the PRT intervention did not include task-specific exercises. In our review, three studies use muscle-isolation (i.e., focusing on one muscle), open kinetic chain exercises such as leg presses, hamstring curls, leg extensions, and calf raises (Buckley et al., 2008; Schilling et al., 2010; Shulman et al., 2013), while two studies used an eccentric resistance training paradigm on a cycle ergometer (Dibble et al., 2006, 2009) and only one study included functional, closed kinetic chain exercises (i.e., squats, stepping up and down, and heel raises) (Allen et al., 2010). It is possible that the non-specific nature of the PRT exercises did not directly train gait-like movement patterns, which limited the potential for gait improvements using outcome measures identified. It may also be possible that the outcome measures used in the studies were not sensitive enough to detect changes in gait and balance that are associated with PRT. Lastly, it could be that the small number of studies involved in this meta-analysis and underpowered studies used were not enough to detect a significant difference between groups (Turner et al., 2013).

In conclusion, our meta-analysis examining the effect of PRT on measures of gait and balance in people with PD found inconclusive evidence to support or refute the use of PRT. Based on our findings and the understanding of the influence of task-specific functional training and balance training for people with PD, it is suggested that PRT should be used in conjunction with balance and task-specific functional training to further improve measures of gait and balance. Finally, our systematic review revealed a dearth of information regarding direct measures of balance and gait, which should be included in future PRT intervention studies.

## Conflict of Interest Statement

The authors declare that the research was conducted in the absence of any commercial or financial relationships that could be construed as a potential conflict of interest.
